# Enhancing the Capacity of Local Health Departments to Address Birth Equity: The Institute for Equity in Birth Outcomes

**DOI:** 10.1007/s10995-021-03135-1

**Published:** 2021-04-30

**Authors:** Vicki Collie-Akers, Sarah Landry, N. Jessica Ehule, Denise Pecha, M. Monica Beltran, Carol Gilbert, Chad Abresch

**Affiliations:** 1 Department of Population Health, University of Kansas Medical Center, 3901 Rainbow Boulevard, MS 1008, Kansas City, KS 66160 USA; 2 University of Nebraska Medical Center, 982170 Nebraska Medical Center, Omaha, NE 68198-2170 USA; 3 Kellogg Foundation, 1 E Michigan Ave, Battle Creek, MI 49017 USA

**Keywords:** Birth outcomes, Equity, Social determinants of health

## Abstract

**Introduction:**

Significant and persistent racial and ethnic disparities exist related to infant mortality and other birth outcomes. Few models exist that aim to prepare organizations to implement essential features, such as community engagement or intervening on social determinants of health.

**Methods:**

Between 2013 and 2015, teams from seven local health departments participated in the Institute for Equity in Birth Outcomes (EI) with the goals of building capacity and implementing changes to address equity in birth outcomes. Four of the teams enrolled in the first cohort (2013–2015), and three enrolled in cohort two (2014–2015). To examine the EI effort and its impact on capacity and implementation of changes, two types of assessments were completed. Capacities of the teams in specific key areas were assessed using “Best Change Process” instruments at the completion of participation in the EI. Teams also documented on an ongoing basis implementation of interventions. The data were analyzed using descriptive statistics and Pearson Correlation tests.

**Results:**

Best Change Process capacity scores were higher in the first cohort than in the second and were highly correlated with implementation of changes (Pearson’s Correlation = 0.838, p = 0.037). Collectively, the teams implemented about 32 new programs, policies, practices, and systems changes aimed at addressing equity in birth outcomes. Most interventions were based on scientific recommendations and local epidemiologic data.

**Discussion:**

The results of the study suggest the EI is a promising approach that may result in strong capacity and ability to implement interventions aimed at addressing equity in birth outcomes.

## Significance Statement

*What is known about this? *Significant and persistent racial and ethnic disparities exist related to infant mortality and other birth outcomes. Despite efforts to address these disparities, few models exist that aim to prepare organizations to implement essential features, such as community engagement or intervening on social determinants of health.

*What this study adds? *This study adds to the field by describing a model for supporting local health departments in developing and implementing changes aimed at addressing equity in birth outcomes and the impact of the model.

## Introduction

Significant disparities in infant mortality persist in the United States. In 2016, the infant mortality rate was 10.75 per 1000 live births among non-Hispanic Black infants and 8.15 per 1000 live births among American Indian or Alaska Native infants. This was compared to 4.63 and 3.63 per 1000 live births among non-Hispanic white and Asian or Pacific Islander infants, respectively(Ely & Driscoll, 2020; Ely et al., 2018). Birth outcomes, such as low or very low birth weight and pre-term delivery rates reflect similar disparities.

Lu and Halfon (Lu & Halfon, 2003) suggested a critical approach to understanding and addressing disparities in birth outcomes: the life-course perspective. They suggest that a life-course perspective acknowledges the cumulative impact of differential risk and protective factors on the reproductive potential of women, including biologic, social, economic, and environmental factors. African American women may encounter more risk factors than white women and Burris and Hacker (Burris & Hacker, 2017) note that a number of factors influence these ongoing health disparities, including income, education, segregation, and psychosocial stressors, including racism.

Several strategies have been suggested to address birth outcomes ranging from efforts to address the clinical care (Hogue & Vasquez, 2002; Ricketts et al., 2005) received by women to increasing reproductive life planning (Malnory & Johnson, 2011). However, the Association of State and Territorial Health Officials’ issue brief (Association of State and Territorial Health Officials, 2012), *Disparities and Inequities in Maternal and Infant Health Outcomes*, concluded “meaningfully engaging with the social determinants of health is a critical component of any comprehensive health equity strategy to improve birth outcomes.” In addition, several sources suggest community engagement is a necessary component to implement sustainable, effective changes in equity (Holden et al., 2016; Ochoa & Nash, 2009; Wallerstein & Duran, 2010).

Despite these recommendations, few examples exist in which addressing social determinants of health, integrating the life course perspective, and robust community engagement are the focus of intervention efforts. The Association of Maternal and Child Health Programs (AMCHP) maintains a database of programs and practices with varying degrees of evidence that measure success as a means of raising up practices to address maternal and child health. AMCHP’s database includes practices referred or submitted for review by researchers or practitioners, which have been vetted and assigned a category describing level of evidence (i.e., cutting-edge, emerging, promising, best). Only two examples of addressing health equity are listed. One example, the Rhode Island Health Equity Zone Initiative (Association of Maternal & Child Health Programs, 2019), appears to include a focus on social determinants of health, use of a life course perspective, and a community engagement component. While providing a robust example of an integrative approach, the model is supported by millions of dollars of funded support and extensive infrastructure and support at the state level, which may not be the context of many communities that seek to address equity in birth outcomes. A gap remains in the literature concerning models for providing assistance to health departments to address equity with a life course perspective. Further, no such model appears in the literature that balances a social determinants perspective with utilization of evidence-based programming. The purpose of this paper is to describe a model for building the capacity of local health departments to use recommended approaches to address equity in birth outcomes.

## Methods

### Intervention

**Fig. 1:**
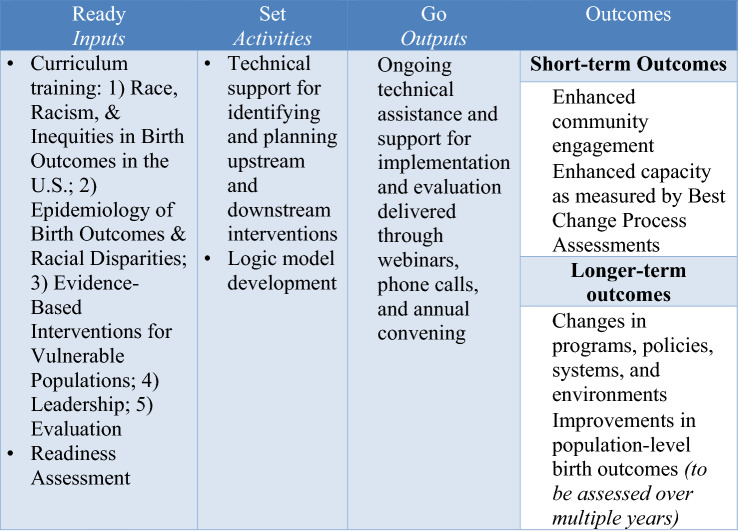
EI intervention phases, components and related outcomes. This figure presents the logic of the EI model. Phases, components, and related outcomes are identified from left to right

CityMatCH is a membership organization of city and county health departments with maternal and child health program areas or departments. CityMatCH’s mission is to strengthen public health leaders and organizations to promote equity and improve the health of urban women, families, and communities. Beginning in 2013, CityMatCH began systematic efforts exploring a model for working with local health departments to address equity in birth outcomes called the Institute for Equity in Birth Outcomes (EI). The EI integrates technical assistance and support in making data-driven decisions and priorities for action. The essential features of the EI intervention are distributed across CityMatCH’s Ready, Set, Go Framework for Health and Healing. The Ready, Set, Go Framework is a practice-based approach to guiding teams through a process of identifying community needs related to equity in birth outcomes, using community engagement approaches to identify and plan for interventions, and ultimately implementation of interventions and initiatives. CityMatCH staff provided technical assistance and support aimed at helping teams engage their communities in a process of identifying and implementing two types of programs. Technical assistance consisted of an annual in-person training, monthly cohort-wide technical assistance webinars, and monthly individual team audio calls throughout the duration of each team’s enrollment in EI. Technical assistance and support focused on working with teams to select two types of programs or initiatives to implement in their communities. The first program type was “upstream” interventions, which focus on the social determinants of health and are designed to impact large-scale policy and systems change. The second program type was “downstream” interventions, which focused on evidence-based programs, capable of rapidly producing (Fig. 1) measurable change and often targeted at individual- and provider-level behavior change (Brownson et al., 2010).

The EI was planned to include multiple cohorts of community teams convened by their local health department, each of which would be enrolled for two to three years. This article assesses the efforts of the first two cohorts, enrolled from 2013 to 2015 and from 2014 to 2015, respectively.

### Recruitment and Inclusion

CityMatCH maintains a membership network of approximately 180 local health departments that receive maternal and child health technical assistance, national representation, and peer-support through the membership organization. To recruit community teams to participate in the EI, CityMatCH distributed recruitment materials via its membership networks. Staff distributed information to partner networks as well. No monetary resources were offered for participation. Recruitment materials described the benefits of participation as availability of training and technical assistance, increased opportunities to collaborate with teams across the country, support for evaluation, and resources for travel to annual meetings. To be considered for inclusion, prospective community teams were required to demonstrate: presence of inequity in birth outcomes at the local level; ability to develop a team co-led by the local health department and a community partner reflective of community members from populations experiencing birth inequities; baseline epidemiology or data analysis capacity; and, support to engage the project by the organizations’ top executives. CityMatCH staff, federal maternal and child health experts, and state and local maternal and child health experts from health departments reviewed applications and selected teams from prospective communities. In cohort one, CityMatCH received 24 applications and reviewers prioritized local health departments with established capacity in the selection of participants. For cohort two, CityMatCH received nine applications and reviewers selected health departments with more variation in capacity. A total of seven community teams were selected to participate in the two cohorts. Cohort one included teams from San Francisco, CA; Baltimore, MD; Dayton & Montgomery County, OH; and Palm Beach County, FL. Cohort two included teams from Orange County, FL; Shelby County, TN; and Seattle & King County, WA.

### Study Design, Measurement, and Analysis

#### Study Design and Analysis

A post-test case study design was used. Data were analyzed using descriptive statistics. Associations between Best Change Process Assessments and program, policy, environmental, and systems changes were explored using a Pearson’s Correlation analysis. Staff analyzed each cohort separately because the cohorts differed on a few key characteristics, particularly the length of time participating in EI.

#### Measurement

Two core measures were used to examine outcomes related to this study. These measures are described in Table 1.

**Table 1:** Description of study outcomes and related measurement

Outcome	Measure	Instrument	Description of instrument:
Capacity to implement changes in communities and systems	Average scores of implementation of tasks	Best change process assessment	Consists of six domains: developing a logic model/ framework for change, developing/ implementing effective interventions, using and assuring technical assistance, documenting progress, making outcomes matter, and building sustainability. Each domain contained 13–24 yes/no questions about the completion of a core task.
Implementation of new programs, policies, systems changes	Discrete instances of new programs, policies, or systems changes	Documentation in the Community Check Box	Online documentation platform which guides users through a series of text boxes and pull-down menus. Community documenters involved with implementation recorded instances of activities and changes as they occurred.

#### Best Change Process Measurement

To examine changes in the readiness and capacity of EI teams to engage in identification and implementation of both upstream and downstream efforts, staff used a Best Change Process Assessment developed by the University of Kansas Center for Community Health and Development (Watson-Thompson et al., 2013). The assessments include questions about the presence of and level of implementation of core tasks associated with enhanced capacity for each of the six best change processes. The primary measures resulting from this tool were percentages of core tasks implemented by each team.

#### Documentation of Implementation of Changes to Programs, Policies, Systems, or Environments

EI teams and evaluation staff documented instances of implementation of efforts aimed at (a) developing a robust collaboration and (b) implementation of the upstream and downstream interventions, as a means of understanding progress in shorter-term outcomes. EI teams were trained to use the Community Check Box Evaluation System (CCB) developed by the University of Kansas Center for Community Health and Development (KU) (Fawcett et al., 2017). Training consisted of providing definitions, coding criteria, examples, and non-examples for key activities and accomplishments. KU staff encouraged teams to document instances development activities (i.e., activities implemented to prepare the team to accomplish its work) and community changes (i.e., new or modified programs, policies, practices, or environmental change) as they were implemented or at least monthly. In a few cases, KU staff conducted interviews to obtain and document information about implementation. KU staff implemented quality assurance procedures throughout data collection. These procedures included checks with the teams for completeness and steps to establish a minimum 90% interobserver agreement for all coding conducted with the data.

Activities documented as community changes (or changes) were required to meet five criteria: (1) a discrete activity which was program, policy, practice, or environmental change, (2) it was new or had been substantively modified, (3) it actually occurred (not merely planned), (4) it was aimed at addressing equity in birth outcomes, and (5) it was brought about by the EI team or EI team partner acting on behalf of the EI team. In addition to documenting instances of changes to programs, policies, systems, or environments, each instance of a change (either new or substantively modified), was characterized by three key characteristics that may be helpful in understanding the dose or intensity of each effort (Collie-Akers et al., 2013; Fawcett et al., 2015). The three characteristics each were scored using standard definitions and scoring criteria to create a low (0.1), medium (0.55), or high (1.0) score. These included: the number and percentage of the priority population reached for each instance (i.e., low: 0–5%, medium: 6–20%, or high: 21% or higher of the priority population reached), the behavior change strategy used (i.e., low: providing information or enhancing skills, medium: enhancing services or support or changing consequences, or high: modifying access, opportunities or barriers or modifying systems), and the duration of the change (i.e., low: one-time event, medium: occurring more than once with a known endate, or high: occurring continuously). These categories are used to assign each change an individual intensity score (ranging from 0 at the lowest to 1 at the highest). Individual intensity scores are summed annually to create a composite annual intensity score for each community. The composite annual intensity score served as a measure for the many programs, policies, practices, and environmental changes any one community implemented. The methods used to measure intensity replicated methodology used to examine the association between community programs and policies and population-level health outcomes (Strauss et al., 2018).

This study complied with prevailing ethical standards. The study was reviewed by the University of Kansas Human Subjects Committee (Institutional Review Board) and determined to not be human subjects’ research.

## Results

### Best Change Process Assessment Scores

Assessments were conducted at the end of each cohort’s engagement in EI. Table 2 contains descriptive data regarding the ratings provided by EI teams.

**Table 2:** Mean percentage of best change processes core tasks completed

	Cohort 1	Cohort 2
	mean percentage of core tasks implemented (std dvtn)	mean percentage of core tasks implemented (std dvtn)
Logic model and planning	.84 (.09)	.68 (.13)
Implementing effective interventions	.90 (.05)	.55 (.39)
Technical assistance	.83 (.16)	.68 (.13)
Documenting progress	.81 (.13)	.35 (.2)
Making outcomes matter	.80 (.09)	.39 (.04)
Sustainability	.73 (.17)	.20 (.12)

On average, Cohort 1 teams had high levels of implementation of all processes. Additionally, 90% of core tasks were completed to implement effective interventions. These core tasks include:

- Engaging community members and other stakeholders in designing the intervention;
- Identifying core components, elements, and modes of delivery for the intervention;
- Evaluating the implementation of the intervention and its contributions towards improving outcomes; and,
- Using the information to celebrate, make adjustments, and assure effectiveness of the intervention.

Over 80% of core tasks related to developing a framework for change, assuring technical assistance, documenting progress, and making outcomes matter to promote activities that improve efforts were implemented across all teams. Teams completed the fewest core tasks in planning for sustainability with approximately 73% of the tasks completed on average.

These results show that teams worked both internally and with community partners to take steps to ensure that strategies to reduce disparities in birth outcomes and infant mortality could be successfully implemented, evaluated, and sustained.

Among the six best change processes, Cohort 2 teams had higher levels of implementation for the processes of developing a logic model and planning (68%), assuring technical assistance (68%), and implementing interventions (55%). Core tasks for these processes include activities such as convening stakeholders to develop a logic model for the effort, using the logic model to guide the work, implementing and evaluating the effort, assessing readiness to use technical assistance, identifying appropriate technical assistance and support providers.

### Changes in Programs, Policies, Environments, and Systems

**Fig. 2:**
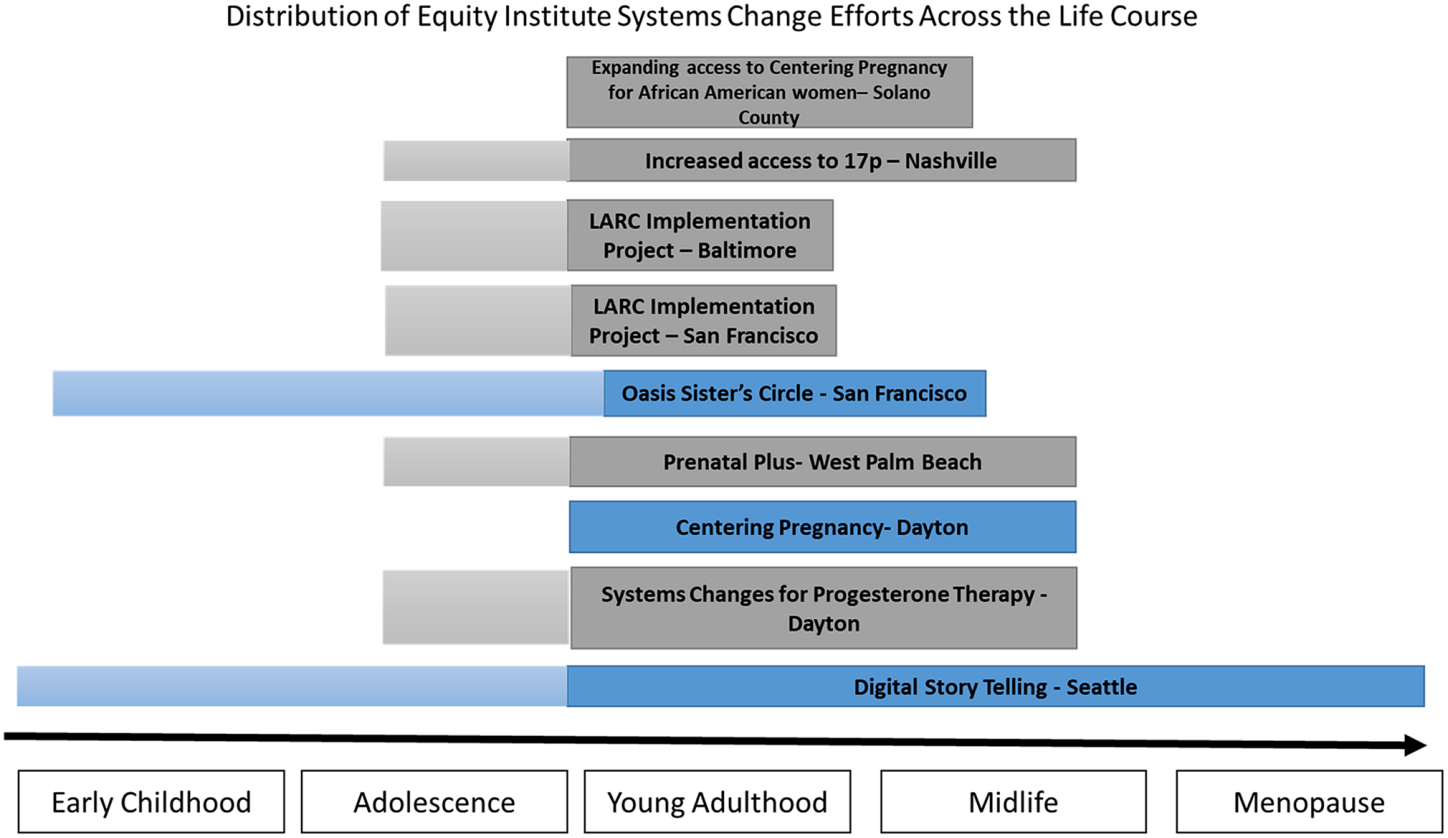
Upstream and downstream interventions across the life course by community. The phases of the life course are laid out along the bottom of the figure from left to right. Each intervention implemented by EI teams was assigned to the parts of the life course in which they intervened (Color figure online)

Participation in the EI required that each team identified both “upstream” and “downstream” interventions to implement. Figure 2 describes the selected interventions and their contribution across a life course.

Items represented in grey were intended to be downstream interventions, and blue items were intended to be upstream interventions.

**Fig. 3:**
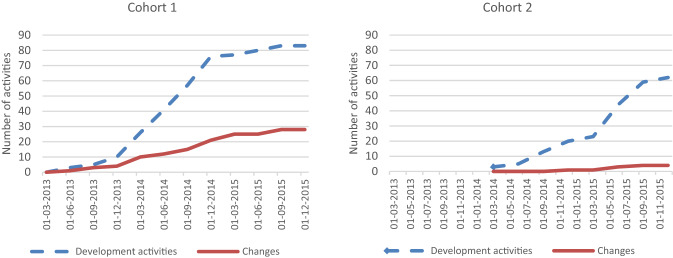
Distribution of development activities and changes distributed over time by cohort. This figure contains two line graphs side-by-side, with cohort 1 on the left and cohort 2 on the right. Each graph displays a dashed cumulative line of development activities and a solid line of community changes implemented over time

To implement the identified upstream and downstream interventions, participating teams conducted a total of 145 development activities teams across both cohorts and 32 changes or new or substantively modified programs, policies, systems, and environments were implemented. Figure 3 reflects how these activities unfolded in each cohort over time.

Illustrative changes to programs, policies, systems, and environments include:

- Implementation of long-acting reversible contraception (LARC) insertion/removal proctoring for clinicians from 2 clinics in San Francisco.
- Implementation of the Healing Ourselves through Peer Empowerment pilot program began for women who have lost or experienced infant mortality.
- Distribution of newly developed FAQs, reimbursement guides, and other documents to support clinics in understanding implementation of the new LARC policy in Baltimore.
- Piloting of reimbursement strategies to assure LARC provision by Baltimore Medical Systems.
- Completion of Choice/Bixby Center training with participating clinic staff in order to improve access to and provide LARC to the target population.
- Piloting of LARC training in Federally Qualified Health Centers (FQHCs) to improve access and provide LARC in Baltimore.
- Provision of Centering Pregnancy® Basic Facilitation Training Workshop to clinic providers.
- Training sonographers at 3 clinics in Dayton to expand availability of services to diagnose short cervix to determine eligibility for progesterone therapy.
- Commencement of the first group Centering Pregnancy® meeting at 5 Rivers Health Center in Dayton.
- Provision of pre-natal case management services through the Prenatal Plus Program
- Piloting of an hour-long “Know Your Choices” workshop that focused on life planning and reviewed all contraceptive methods using a best-practice tiered- approach, presented birth control as a tool for achieving life goals and linked participants to clinic services.

The 32 changes contributed to the implementation of nine distinct interventions. Of the nine interventions, seven had a known-evidence-base, while two were driven by community or expert influence.

In total, the 32 changes had a direct impact or reach on 10,470 people in the participating communities (Cohort 1 = 8786, Cohort 2 = 1684). Documentation efforts suggest that the vast majority of these efforts were focused entirely on community residents from populations with longstanding disproportionately poor birth outcomes. Cohort 1 teams implemented more changes (n = 28), which had broader reach. The intensity of efforts brought about by communities differed by community and by cohort. The average intensity of community changes was 0.28 for Cohort 1 teams and 0.59 for Cohort 2 teams. The average composite intensity (for all changes combined) was 2.76 for Cohort 1 teams and 1.22 for Cohort 2 teams. This suggests that the overall number of changes elevated the average composite scores for Cohort 1, while the lower number of changes in Cohort 2 created lower scores despite higher individual change scores.

Notably the number of community changes brought about by the sites were strongly, positively associated with average ratings provided in the completion of the Best Change Process assessments. A Pearson’s Correlation showed a 0.838 association (p = 0.037). This is strongly suggestive that assuring the development of teams’ capacity will support the implementation of more change in communities.

## Discussion and Conclusions

These findings suggest that a model in which technical assistance, training, and support are provided to encourage teams of local health departments and other collaborators to address equity in birth outcomes can result in increased capacity of such teams and the implementation of program, policy, systems, and environmental changes. Communities engaged in the EI were successfully able to implement change that had wide reach within communities experiencing disproportionately poor birth outcomes. Communities made important and meaningful community and system changes that were directly related to their defined strategies, such as improved provider capacity to provide LARC, and improved access to prenatal care through expansion or implementation of new programs.

The number of community changes brought about by sites was strongly, positively associated with the average ratings provided in the completion of the Best Change Processes assessments. This is strongly suggestive that assuring the development of teams’ capacity will support the implementation of more change in communities. Community engagement is valued and key to supporting the work. Each team described broad cross-sector representation. Additionally, teams described some level of community participation in one of four key domains: leadership, planning, implementation, and monitoring and documenting outcomes. In some cases, teams reported a desire to increase participation where possible. Further, each team reported that either new or enhanced partnerships were developed and were viewed as contributing to sustainability of the work.

Despite relatively equal focus during training and technical assistance regarding upstream and downstream interventions, the participating communities implemented far more changes related to downstream interventions. This may suggest that communities have more familiarity and support for downstream efforts. Conversely, upstream efforts regarding social and economic policy or systems changes may take longer than the study period to plan and execute.

Notably, community teams participating in Cohort 1 implemented more change than in Cohort 2. Although implementing more changes in total, the changes implemented by Cohort 1 had lower average intensity than Cohort 2. Conversely, the cumulative average intensity for Cohort 1 was much higher than Cohort 2. Several factors may have influenced these outcomes. Chief among these, the duration of engagement for Cohort 1 was one year longer than Cohort 2. Also, based on a review of the teams’ initial applications, three of the Cohort 1 teams had already had a history of other collaborative or community engaged efforts to address health disparities in birth outcomes among specific racial or ethnic groups underway. While these efforts were discrete from those attributed to engagement in EI and were not necessarily conducted with an equity focus, the community teams may have been more prepared to collaborate and implement other approaches. An indication of this enhanced readiness may be seen in the amount of time it took teams to bring about community changes. Cohort 1 teams took an average of 5 months to initiate changes, while it took Cohort 2 teams an average of 17 months to initiate change efforts.

Several challenges and strengths can be noted with this study. The study used a post-test design and would have been strengthened by a more robust design. For example, pre-intervention measures or the use of a comparison group may have provided more evidence of the impact of the effort or minimized concerns that the selected teams were more able to implement these changes. The intent of the EI was to be inclusive of local health departments and their partners with varying degrees of experience in addressing birth outcomes. Some of the success in bringing about changes may have been influenced by the capacity the teams had when beginning their engagement in EI. Despite these limitations, the EI is unique its attempt to drive communities to consider upstream and downstream interventions concurrently. Indeed, the inclusion of efforts to address upstream influences of inequity in birth outcomes is a novel approach. Lastly, the pairing of making data- and community-driven decisions about priorities and decision-making is a critical approach to making relevant and lasting changes in communities.

The EI shows promise in leading to changes in programs, policies, systems, and environments that may have an impact on birth outcomes. Ongoing evaluation activities and data collection are occurring to better understand the impact on birth outcomes that take longer to change. The findings of this study offer promising information for public health practitioners addressing equity in birth outcomes and provide clarity on the training and support needed to aid community partnerships in their efforts.

Changes in disparity of birth outcomes take considerable time and it is often challenging to observe significant change in long-term outcomes within a 2 year time frame. However, short and intermediate outcomes showed positive impact in the capacity of participating health departments to implement change and actual implementation of change, indicating that sustaining and expanding the work of participating teams is likely to have a positive impact on the planning and implementation of additional data- and community-driven programs designed to reduce inequities in birth outcomes. The authors wish to thank the Equity Institute teams featured in this article. Their work to advance equity in birth outcomes serves as a model and inspiration to the field. In addition, the authors wish to extend gratitude to the past and current CityMatCH staff who provided support to EI efforts.

## Data Availability

Data are available upon request.

## References

[CR1] Association of State and Territorial Health Officials (Producer) (2012). Disparities and inequities in maternal and infant health outcomes. [Issue Brief]

[CR2] Association of maternal and child health programs. (2019). Innovation station practice summary and implementation guidance: Rhode island health equity zones. Retrieved from http://www.amchp.org/programsandtopics/BestPractices/InnovationStation/ISDocs/Health%20Equity%20Zones.pdf

[CR100] Brownson, R. C., Seiler, R., & Eyler, A. A. (2010). Measuring the impact of public health policy. *Preventing Chronic Disease*, *7*(4), A77 PMC2901575 20550835

[CR3] Burris HH, Hacker MR (2017). Birth outcome racial disparities: A result of intersecting social and environmental factors. Seminars in Perinatology.

[CR200] Collie-Akers, V. L., Fawcett, S. B., & Schultz, J. A. (2013). Measuring progress of collaborative action in a community health effort. *Revista Panamericana de Salud Pública*, *34*(6), 422–428 PMC6352719 24569971

[CR4] Ely, D., & Driscoll, A. (2020). *Infant Mortality in the United States, 2018: Data from the period linked birth/infant death file*. Hyattsville, MD. Retrieved from https://www.cdc.gov/nchs/data/nvsr/nvsr69/NVSR-69-7-508.pdf 32730740

[CR5] Ely, D., Driscoll, A., & Mathews, T. (2018). *Infant mortality by ageat death in the United States, 2016*. Hyattsville, MD. Retrieved from https://www.cdc.gov/nchs/data/databriefs/db326-h.pdf 30475688

[CR400] Fawcett, S. B., Collie-Akers, V. L., Schultz, J. A., & Kelley, M. (2015). Measuring community programs and policies in the healthy communities study. *American Journal of Preventive Medicine*, *49*(4), 636–641 10.1016/j.amepre.2015.06.027 PMC4575764 26384934

[CR300] Fawcett, S., Schultz, J., Collie-Akers, V. L., Holt, C., Watson-Thompson, J., & Francisco, V. (2017). Participatory monitoring and evaluation of community health initiatives using the Community Check Box evaluation system. In N. Wallerstein, B. Duran, & M. Minkler (Eds.), *Community-based participatory research for health* (3rd ed.). San Francisco, CA: Wiley

[CR6] Hogue CJ, Vasquez C (2002). Toward a strategic approach for reducing disparities in infant mortality. American Journal of Public Health.

[CR7] Holden K, Akintobi T, Hopkins J, Belton A, McGregor B, Blanks S, Wrenn G (2016). Community engaged leadership to advance health equity and build healthier communities. Social Sciences (Basel).

[CR8] Lu, M. C., & Halfon, N. (2003). Racial and ethnic disparities in birth outcomes: a life-course perspective. *Maternal and child health journal*,* 7*(1), 13–30. Retrieved from https://www.ncbi.nlm.nih.gov/pubmed/12710797 10.1023/a:1022537516969 12710797

[CR9] Malnory ME, Johnson TS (2011). The reproductive life plan as a strategy to decrease poor birth outcomes. Journal of Obstetric, Gynecologic & Neonatal Nursing.

[CR10] Ochoa ER, Nash C (2009). Community engagement and its impact on child health disparities: Building blocks, examples, and resources. Pediatrics.

[CR11] Ricketts SA, Murray EK, Schwalberg R (2005). Reducing low birthweight by resolving risks: Results from Colorado’s prenatal plus program. American Journal of Public Health.

[CR12] Strauss WJ, Nagaraja J, Landgraf AJ, Arteaga SS, Fawcett SB, Ritchie LD, John LV, Gregoriou M, Frongillo EA, Loria CM, Weber SA, Collie-Akers VL, McIver KL, Schultz J, Sagatov RDF, Leifer ES, Webb K, Pate RR, Healthy Communities Study Team (2018). The longitudinal relationship between community programmes and policies to prevent childhood obesity and BMI in children: The healthy communities study. Pediatric Obesity.

[CR13] Wallerstein N, Duran B (2010). Community-based participatory research contributions to intervention research: the intersection of science and practice to improve health equity. American Journal of Public Health.

[CR14] Watson-Thompson J, Woods NK, Schober DJ, Schultz JA (2013). Enhancing the capacity of substance abuse prevention coalitions through training and technical assistance. Journal of Prevention & Intervention in the Community.

